# Withdrawn

**DOI:** 10.1111/pbi.13748

**Published:** 2023-07-28

**Withdrawal:** Wu, H., Hu, Q., Ai, F., Zhang, J., Kang, M., Lin, H., Xu, Z., Dong, G., Ma, T. and Liu, J. (2023), A chromosome‐level genome assembly for the wild kiwifruit *Actinidia kolomikta* provides insights into canker resistance and fruit development. Plant Biotechnology Journal. Accepted Author Manuscript. https://doi.org/10.1111/pbi.13748. The above article, published online on 17 November 2021 in Wiley Online Library (wileyonlinelibrary.com), has been withdrawn by agreement between the authors, the journal's Editor‐in‐Chief, Johnathan Napier, the Society for Experimental Biology, Association of Applied Biologists and John Wiley & Sons Ltd. The withdrawal has been agreed because the species described in the article was disputably misidentified.

